# The 13-year observation of hip fracture in Poland—worrying trend and prognosis for the future

**DOI:** 10.1007/s40520-017-0747-2

**Published:** 2017-03-28

**Authors:** Robert Wilk, Michał Skrzypek, Małgorzata Kowalska, Damian Kusz, Bogdan Koczy, Piotr Zagórski, Wojciech Pluskiewicz

**Affiliations:** 1 Department of Orthopaedics and Traumatology, School of Medicine in Katowice, Medical University of Silesia in Katowice, Ziołowa 45/47 Street, 40-635 Katowice, Poland; 2 Department of Biostatistics, School of Public Health in Bytom, Medical University of Silesia in Katowice, Bytom, Poland; 3 Department of Epidemiology, School of Medicine in Katowice, Medical University of Silesia in Katowice, Katowice, Poland; 4 Department of Trauma and Orthopaedics, District Hospital of Orthopaedics and Trauma Surgery, Piekary Śląskie, Poland; 5 Department of Orthopaedics and Traumatology, Sport-Clinic, Żory, Poland; 6 Metabolic Bone Diseases Unit, Department and Clinic of Internal Diseases, Diabetology and Nephrology, School of Medicine with the Division of Dentistry in Zabrze, Medical University of Silesia in Katowice, Zabrze, Poland

**Keywords:** Crude and standardized incidence, Epidemiology, Hip fracture, Osteoporosis

## Abstract

**Introduction:**

Hip fractures are often considered to be one of the most common osteoporotic fractures. In our previous study, we noted the increasing trend in the total number of hip fractures as well as crude and standardized rates, for both women and men. This observation enabled us to delve deeper into the study of osteoporotic fractures.

**Methods:**

Hospital records between 1.01.2002 and 31.12.2014 with ICD-10 codes S72.0. S72.1 and S72.2 (femoral neck. intertrochanteric, subtrochanteric, and inter and subtrochanteric fracture) were analysed. All fractures occurred in citizens who lived in the district Tarnowskie Góry and the city of Piekary Śląskie aged 50 years and more.

**Results:**

1507 fragility hip fractures (400 in men, and 1107 in women) were registered. The rates increase in both sexes was still observed. The tendency to sustain fractures was lower in female (29.3%) than in the male population (63.6%). We observed a lower increase in urban (35.8%) population when compared to rural (40.8%) population. Incidence rate ratios for female gender were 1.89 (95% CI 1.65–2.18). The rates in 2014 were as follows: crude rate of 216.2 (men 140.9; women 276.5) and standardized 183.9 (131.6 and 219.4, respectively). This observation allowed as to project a total crude rate of 467.2 (men 329.6; women 584.7) for the year 2050.

**Conclusions:**

The number of osteoporotic hip fractures in Polish men and women is still relatively low, but the epidemiological situation is getting worse. The over 13 years of follow-up demonstrated that the trend to increase in total number of hip fractures for men and women is still observed. This prognosis is of a major concern.

## Introduction

Osteoporosis is one of the most serious problems in the aging populations because of the lack of effective prophylaxis and poor outcomes of treatment [1, 2]. In most cases, osteoporosis leads to low-energy fractures (fragility fractures). According to the newest estimation, the expected number of people at high risk of osteoporotic fracture will be doubled in the year 2040 [3]. One of the most common osteoporotic fracture is hip fracture [4]. It usually leads to a significant burden on the patient and close family because of the need to support, generate serious financial, and social costs. The fracture is also related to high mortality of 26.5% in the first year [5]. According to the “world-wide projections for hip fracture” starting from 1997, the number of hip fracture will approximately double by the year 2025 and will more than triple by 2050 [6]. The observed data confirm the increase in hip fractures [7]. Some groups of scientists developed a special programs and created new indexes useful in the prophylaxis [8, 9]. Fortunately, nowadays, there are reports that in some countries, the incidence rate of hip fracture is decreasing [7, 10, 11].

In our previous study, we had observed (in Poland) an increasing trend in total number of hip fractures as well as crude and standardized rate of hip fracture for women and especially for men [12]. The results were alarming, so we decided to continue our observation of the citizens in the district of Tarnowskie Góry and the city of Piekary Śląskie and predict number of fracture and incidence rates in the future.

## Methods

### Study population

The study area spans the district of Tarnowskie Góry and the city of Piekary Śląskie. This area is located in the historical region of Upper Silesia (Southern Poland) and comprises of rural and urban territories (we used the administrative division to divide the areas into rural and urban ones). The rural–urban ratio is similar to the one among the total population of Poland [13]. In 13 years of our observation, the number of peoples aged 50 years or more increased by 23.97% (25.09% for men and 23.08% for women). However, the total number of citizens decreased by 2.30%. The total study population (aged 50 and over) in 2014 was 74.947. These data confirm the aging process characteristic for the Polish population [13, 14]. In 2014 (among the polish population), the life expectancy was 73.06 years for men and 81.14 years for women [13]. The population was 100% Caucasian.

There is only one orthopaedic hospital in the study area where all patients with suspected fractures are managed. Therefore, all of the patients were treated in one institution—Dr. J. Daab Regional Hospital of Trauma Surgery in Piekary Śląskie. All case records of the patients aged 50 years or more between January 1, 2002 and December 31, 2014 with code of the International Classification of Diseases ICD-10: S72.0; S72.1; and S72.2 (cervical, intertrochanteric, subtrochanteric, and inter and subtrochanteric fracture) were studied [15].

### Assessments and exclusion criteria

Only fragility fractures (caused by the fall from a standing height or less) of patients living in the study area were analysed. Data were obtained from personal interview and based on the ICD-10 code (V01-Y98) of external causes of morbidity and mortality [15]. Patients living outside of studied area and those with high energy fractures (e.g., violent trauma, automobile accidents, falls from more than standing height, etc.) were excluded from the analysis. The diagnosis was based on radiographs or CT if the fracture was not evident on X-ray. Every duplicate record or data in case of readmission were excluded from the final data set. For estimating all necessary rates, we first calculated the crude ones for hip fracture *N*/100,000 total population of studied area, and then proceeded to calculate the crude specific rates for male and female population or separately for urban and rural areas.

### Statistical analyses

The calculations were made based on the assumptions adopted in the previous study [12]. Current and projected number of subjects according to gender, age, and place of residence were obtained from the local database available in the Central Statistical Office in Warsaw [13]. We then used the directly standardized procedure to calculate standardized rates for hip fracture based on principles adopted in epidemiology. We used the Segi “world” population as the standard population. Direct standardization yields a standardized rate, which is a weighted average of the age-specific rates, for each of the populations to be compared. The 95% confidence intervals (CIs) were also calculated, assuming Poisson distribution and gamma distribution when the number of incidences was small. The trend for fracture incidences was analysed by the means of multiple Poisson regression model incorporating age and gender as confounders. Incidence rate ratios (IRR) to the first year of registration (2002) were calculated with adjustment for overdispersion (which may occur when observed variance exceeds the variance obtained by theoretical model). We have also computed 95% CIs of IRR using the profile-likelihood function. The estimation methods and relevant bibliography have exactly been described in previously publication [12]. Differences between the ages of patients in particular group (outdoor/indoor fracture) were assessed by the Mann–Whitney *U* test. The fractures sustained at home or in institutions like nursing home, etc., were classified as indoor fractures. The rest of the fracture sustained outside home (on the street etc.) was classified as outdoor ones. Interpretation of statistical significance was based on *α* = 0.05 criterion. Linear regression model was used to assess the predicted number of hip fractures. The actual hip fracture rates in the years 2002–2014 were considered as the dependent variable, whereas year, sex, age group, and their interactions were designated as independent variables. All analyses were performed by means of SAS 9.4 (SAS Institute Inc., Gary, NC, USA).

## Results

1507 patients with fragility hip fracture were registered in from 2002 to 2014, in the district of Tarnowskie Góry and the city of Piekary Śląskie (average age 78.29 ± 10.28 years, median of age 80 years). The study group was mostly women (*N* = 1107; 73.46%). Studied female population was older than their male counterpart (mean age 79.98 ± 9.30 vs. 73.60 ± 11.36 years, respectively; *p* < 0.01). Most patients lived in the urban area (*N* = 1167; 77.44%). The percentage of women living in the urban area was slightly higher (73.7% in urban and 72.7% in rural). The average age of people living in the urban territory was slightly higher than the one of those living in rural one (78.4 ± 10.3 and 77.8 ± 10.3 years, respectively, *p* = 0.4). Detailed descriptive statistics are presented in Table 1.

**Table 1:** Descriptive statistics of study group

	Number of cases and % of study group	Mean age ± SD (years)	Median (IQR) (years)
Total	*N* = 1507 (100%)	78.29 ± 10.28	80 (13)
Male	*N* = 400 (26.54%)	73.60 ± 11.36	74 (17)
Female	*N* = 1107 (73.46%)	79.98 ± 9.30	81 (12)
Urban	*N* = 1167 (77.44%)	78.42 ± 10.27	80 (13)
Rural	*N* = 340 (22.56%)	77.83 ± 10.31	80 (14)
Indoor	*N* = 1221 (81.02%)	79.78 ± 9.62	81 (12)
Outdoor	*N* = 286 (18.98%)	71.90 ± 10.56	74 (16)

*SD* standard deviation, *IQR* inter quartile range

Table 2 details the number, crude, and standardized incidence of hip fracture with their 95% CI in particular years separately for total, male, and female population which are presented. The number of fractures is systematically increasing from the starting year of the study period (from 78 to 162 cases) both in men and women (3.6- and 1.8-fold, respectively, comparing to the first year—2002). IRR for female gender was 1.89 (95% CI 1.65–2.18) and was statistically significant. Compared to our previous study, the increase was much lower in female (29.3%) than in male population (63.6%). Similarly, a slight lower increase was observed in the urban (35.8%) than in the rural (40.8%) population [12]. The calculated standardized coefficients are lower than crude.

**Table 2:** Number of registered hip fracture, crude, and standardized rate in population of Tarnowskie Góry and Piekary Śląskie

Year		Total	Male	Female	Urban	Rural
2002	Number	78	13	65	60	18
	Crude rate per 100,000 population	129.0 (102.0–161.0)	48.8 (26.0–83.4)	192.4 (148.5–245.2)	126.5 (96.5–162.8)	138.2 (81.9–218.4)
	Standardized rate per 100,000 population	114.9 (90.7–143.5)	47.8 (25.4–81.8)	155.2 (119.5–198.1)	113.3 (86.4–145.9)	124.8 (73.2–198.8)
2003	Number	82	25	57	64	18
	Crude rate per 100,000 population	132.4 (105.3–164.4)	91.4 (59.2–135.0)	164.8 (124.8–213.6)	131.7 (101.4–168.1)	135.3 (80.2–213.8)
	Standardized rate per 100,000 population	118.1 (93.8–146.7)	90.4 (58.5–133.4)	136.9 (103.4–177.9)	119.8 (92.2–153.1)	113.0 (66.5–179.5)
2004	Number	89	25	64	67	22
	Crude rate per 100,000 population	140.5 (112.8–172.9)	89.3 (57.8–131.9)	181.0 (139.4–231.1)	134.5 (104.2–170.8)	162.6 (101.9–246.3)
	Standardized rate per 100,000 population	124.5 (99.9–153.4)	88.6 (57.3–130.9)	145.6 (111.9–186.2)	121.8 (94.3–154.9)	132.4 (82.6–201.2)
2005	Number	99	20	79	73	26
	Crude rate per 100,000 population	152.3 (123.8–185.4)	69.3 (42.3–107.1)	218.4 (172.9–272.2)	142.7 (111.8–179.4)	187.7 (122.6–275.0)
	Standardized rate per 100,000 population	133.8 (108.6–163.2)	67.8 (41.3–104.9)	178.8 (141.1–223.5)	127.9 (100.1–161.0)	152.6 (99.0–224.7)
2006	Number	90	20	70	71	19
	Crude rate per 100,000 population	135.4 (108.9–166.4)	67.8 (41.4–104.8)	189.3 (147.6–239.2)	135.8 (106.0–171.3)	134.1 (80.7–209.3)
	Standardized rate per 100,000 population	118.4 (94.9–145.8)	66.0 (40.1–102.4)	152.8 (118.7–193.7)	120.5 (93.9–152.3)	112.2 (66.7–176.9)
2007	Number	116	30	86	88	28
	Crude rate per 100,000 population	170.6 (141.0–204.6)	99.5 (67.1–142.0)	227.3 (181.8–280.7)	164.3 (131.8–202.4)	194.0 (128.9–280.4)
	Standardized rate per 100,000 population	114.9 (90.7–143.5)	47.8 (25.4–81.8)	155.2 (119.5–198.1)	113.3 (86.4–145.9)	124.8 (73.2–198.8)
2008	Number	105	31	74	87	18
	Crude rate per 100,000 population	151.2 (123.7–183.1)	100.3 (68.2–142.4)	192.0 (150.8–241.1)	159.2 (127.5–196.3)	121.8 (72.2–192.5)
	Standardized rate per 100,000 population	130.7 (106.6–158.4)	97.3 (65.8–138.6)	151.6 (118.8–190.6)	139.6 (111.6–172.5)	99.6 (58.3–158.9)
2009	Number	144	39	105	114	30
	Crude rate per 100,000 population	203.6 (171.7–239.7)	123.9 (88.1–169.4)	267.5 (218.8–323.9)	205.2 (169.3–246.5)	197.9 (133.5–282.5)
	Standardized rate per 100,000 population	182.6 (153.6–215.5)	127.3 (90.1–174.8)	214.9 (175.5–260.6)	185.0 (152.3–222.6)	177.0 (117.5–256.0)
2010	Number	134	37	97	98	36
	Crude rate per 100,000 population	186.6 (156.3–221.0)	115.5 (81.3–158.9)	243.8 (197.7–297.4)	173.4 (140.8–211.3)	235.3 (164.8–325.7)
	Standardized rate per 100,000 population	165.8 (138.8–196.5)	112.3 (79.0–154.8)	201.8 (163.3–246.6)	154.6 (125.5–188.5)	205.9 (143.4–286.4)
2011	Number	133	36	97	100	33
	Crude rate per 100,000 population	182.8 (153.1–216.7)	111.2 (77.9–153.9)	240.3 (194.9–293.2)	175.0 (142.4–212.8)	211.7 (145.7–297.3)
	Standardized rate per 100,000 population	162.8 (136.3–193.1)	108.4 (75.9–150.1)	195.6 (158.5–238.9)	157.8 (128.3–192.1)	180.5 (124.0–253.8)
2012	Number	135	43	92	116	19
	Crude rate per 100,000 population	183.4 (153.8–217.1)	131.3 (95.0–176.9)	225.2 (181.6–276.1)	201.0 (166.1–241.1)	119.6 (72.0–186.8)
	Standardized rate per 100,000 population	165.4 (138.5–195.9)	129.5 (93.7–174.5)	188.7 (151.7–232.1)	182.4 (150.5–219.0)	106.2 (63.6–166.4)
2013	Number	140	34	106	108	32
	Crude rate per 100,000 population	188.5 (158.6–222.4)	102.9 (71.3–143.8)	257.1 (210.5–311.0)	185.4 (152.1–223.9)	199.8 (136.6–282.0)
	Standardized rate per 100,000 population	163.3 (173.3–192.8)	99.0 (68.5–138.4)	204.2 (167.1–247.1)	161.7 (132.5–195.4)	170.1 (116.2–240.4)
2014	Number	162	47	115	121	41
	Crude rate per 100,000 population	216.2 (184.1–252.1)	140.9 (103.5–187.4)	276.5 (228.3–331.9)	206.3 (171.2–246.5)	215.6 (180.6–341.3)
	Standardized rate per 100,000 population	183.9 (156.6–214.7)	131.6 (96.7–175.1)	219.4 (180.8–263.9)	176.6 (146.4–211.2)	210.0 (150.6–285.1)

Silesian voivodship (Poland) in years 2002–2014 by gender and place of resident (95% CI in the bracket)

**Fig. 1:**
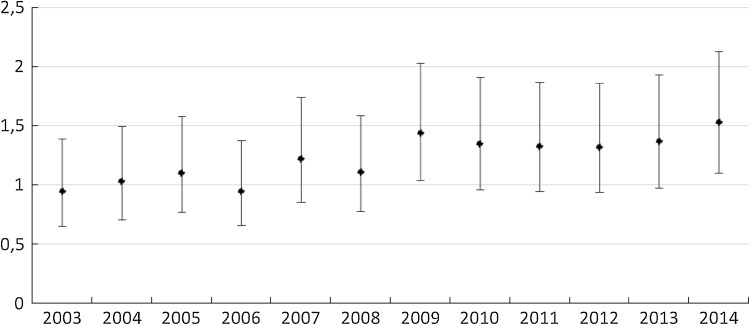
Incidence rate ratio relative to year 2002 and its 95% CI for hip fracture in district Tarnowskie Góry and city Piekary Śląskie. Silesian voivodeship in the period 2003–2014

**Fig. 2:**
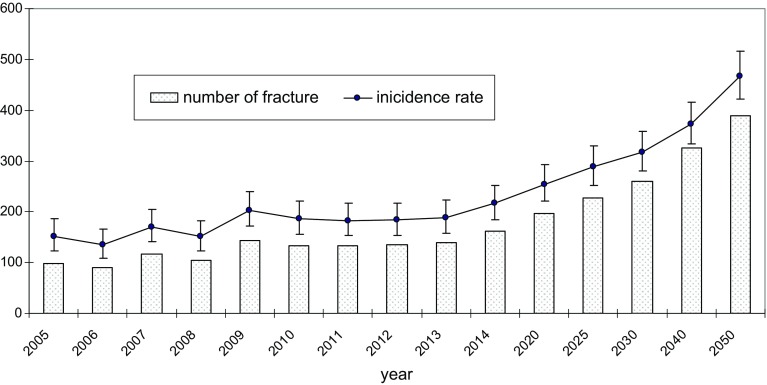
Fragility hip fracture projection in study area to year 2050 for total population (number of fracture and incidence rate with 95% CI)

Most of the incidents occurred indoor. Only about 19% of them happened outdoor (Table 1). The patients who had suffered fractures outdoors were younger (71.9 comparing to 79.8, *p* < 0.01), with the detailed data shown for the particular age in Table 3. The situation is the same in both sexes (men 67.8 and 76.0, women 74.8 and 80.9, respectively, *p* < 0.01). Men were more likely than women to sustain outdoor fracture (29.5 and 15.2% respectively, *p* < 0.01). In rural and urban areas, the rate of fractures outdoor to indoor was similar, 80.88 and 81.06%, respectively. Patients who sustained outdoor fracture were also younger (72.4 and 79.1; 71.7 and 80.0, respectively, *p* < 0.01). During the study period, we observed the increasing tendency of “indoor fracture”. It was evident that outdoor events remained the same and the difference between the studied years was not significant (*p* = 0.09 for Chi-squared test). Figure 1 shows the trend for IRR relative to the year 2002 and its 95% CI for hip fracture. A systematic increase in IRR compared to the first year of registration (2002) should be noted. In the last 4 years, the ratio was always above 1.3 with peak in 2014. The highest value was obtained in 2014—1.53 (95% CI 1.1–2.1). We estimate that in a period of 50 years, there will be a fivefold increase in the number of hip fractures in study area (tenfold increase in men and fourfold in women). From 78 fractures in 2002 to 390 in 2050. In 2050, the crude rate is projected to be 467.2/100,000 (95% CI 422.0–515.9) for all population and 329.6 (95% CI 274.8–392.2) and 584.7 (95% CI 516.2–659.7) for men and women, respectively (Fig. 2; Table 4).

**Table 3:** Differences between age of patients with diagnosed fracture in group defined by place of case, study period 2002–2014

Year	Place	Number of fractures	Age of patients (years)			Results of Mann–Whitney *U* test (*p*)
			Mean	SD	Median	
2002	Outdoor	12	72.92	11.51	74.00	0.1
	Indoor	66	77.94	9.90	79.00	
2003	Outdoor	23	71.91	9.28	76.00	0.1
	Indoor	59	76.54	9.79	77.00	
2004	Outdoor	17	70.41	10.40	70.00	<0.01
	Indoor	72	79.56	8.26	80.50	
2005	Outdoor	26	74.38	8.42	74.50	0.08
	Indoor	73	78.30	9.73	79.00	
2006	Outdoor	23	71.30	9.25	72.00	0.03
	Indoor	67	76.66	10.04	78.00	
2007	Outdoor	25	73.52	11.17	74.00	<0.01
	Indoor	91	80.00	8.92	81.00	
2008	Outdoor	21	75.90	9.02	76.00	0.02
	Indoor	84	81.13	10.01	83.50	
2009	Outdoor	24	68.21	12.52	65.50	<0.01
	Indoor	120	78.88	10.45	80.00	
2010	Outdoor	25	71.80	12.30	74.00	<0.01
	Indoor	109	79.97	9.34	81.00	
2011	Outdoor	18	68.22	12.42	66.00	<0.01
	Indoor	115	80.28	8.82	82.00	
2012	Outdoor	19	70.74	12.08	72.00	<0.01
	Indoor	116	78.99	10.47	81.00	
2013	Outdoor	29	72.76	9.84	76.00	<0.01
	Indoor	111	82.26	9.08	84.00	
2014	Outdoor	24	71.54	9.25	70.50	<0.01
	Indoor	138	82.41	8.80	84.00

**Table 4:** Projected number and crude rates of hip fractures in study area for men and women (95% CI in the bracket)

Population	2020	2025	2030	2040	2050
Men					
Number	59	70	82	104	127
Crude rate	172.7 (131.4–222.8)	200.3 (156.2–253.0)	222.2 (176.6–276.0)	263.4 (215.3–319.1)	329.6 (274.8–392.2)
Women					
Number	137	158	179	221	263
Crude rate	320.7 (269.2–379.2)	360.1 (306.1–420.9)	393.3 (337.8–455.3)	463.5 (404.4–528.8)	584.7 (516.2–659.7)

## Discussion

The most important finding of the current long-term observation is a constant increasing trend of hip fracture incidence. The results confirm our previous observation [12]. In comparison to the year 2002, the IRR increased to 1.53 (95% CI 1.1–2.1) in 2014. In 2002, there were only 78 fractures; however, in 2014, the number approximately doubled to 162. From 2010 (the last year of our previous observation), the system of fall prevention and osteoporosis treatment in Poland has not changed. Current estimation rates are in accordance with this hypothesis. Nowadays, despite the increase in the total number of older people and the number of fractures, there are still no clear, widely accepted national standards for osteoporosis prevention. In the Upper Silesia, there are only four osteoporosis clinics (for 4593 358 inhabitants; 902,781 above 65) paid by the National Health Fund which causes a significant lengthening of waiting lists for medical advice or specialist treatment [13, 14].

The results of the current, long-term follow-up, and our prognosis show the urgency to create orthopaedic and traumatology departments for the older inhabitants and to necessitate their supervision by geriatricians. After stay in the orthogeriatic department, patients should be transfer direct to the rehabilitation department. Nowadays, in most cases, after surgery, they are discharged home directly. It causes worse outcomes and a heavy burden for the family, whom in this instance must generate and assume both indirect (dismissal from work, deficiency of rest, and mental stress) and direct (housekeeper employment, purchase a variety of house equipment, and transport of the patient to a rehabilitation center) costs. Analyzing the data may show a malfunction of the health care system in our country, which must first be recognized that the problem of fractures in older people is a challenge for public health system in Poland [16].

Poland, when compared to other countries, the value of standardized incidence is still slightly less, especially than those observed in other European countries like Austria, Germany, or Italy. We have noted a systematic increase from year to year, while others record its slow decrease. For example, age-standardized hip fracture incidence rates among Austrians aged 50+ have began to decrease from 2006 (from value 491/100,000 to value 456/100,000 in 2008) [17]. In France, the decreasing trend was observed between the years 2002–2013 where they had noted the decline from 6929 to 5987 per 1,000,000 [18]. In our neighbouring Germany, there was no significant trend during the observation period 1995–2010 [19]. However, the crude incidences was estimated as 121.7 (95% CI 120.9–122.4) per 100,000 population in 1995 and 156.9 (95% CI 156.1–157.7) per 100,000 in 2010 [19]. The situation has improved when compared to the previous observation from 1995 to 2004. There was a statistically significant increase in hip fracture incidence of about 1% per year (IRR 1.01, 95% CI 1.00–1.01) [20]. The total increase in study period was 5% (IRR 1.05, 1.02–1.07) [20]. A similar situation in Poland is also observed in Spain, between 1997 and 2010, where the number of hip fracture and the incidence rate has still increased [21]. The crude incidence rate changed from 259.24 to 664.79 in 1997 to 325.30 and 766.37 in 2010, for men and women respectively (the study group consisted of patients 65+ years) [21]. The increasing trend of number of hip fractures was also confirmed by Foronda who analysed data in different regions of Spain [22]. In general, the number of hip fractures increases, but in some countries, the trend of incidence rate is decreasing [7, 10].

In 2010, the crude rate was 186.6/100,000 (men 115.5 and women 243.8); however, standardized one was 165.8 (112.3 and 201.8) [12]. Current calculations show that in 2014, the rates are much higher: crude amounted 216.2 (men 140.9, women 276.5) and standardized one 183.9 (131.6 and 219.4, respectively).

Kanis in his study showed standardized annual hip fracture rates from 63 countries [23]. Comparing our previous calculations with the new Polish rate, we have observed a low incidence of hip fracture. In Europe, only Croatia has lower rate and other countries have either similar rates like Spain and The Netherlands or much higher like Denmark, Sweden, and Austria [23].

According to our observation, the obtained results suggest that most of the fractures had taken place in the indoors, either at home or institution like nursing home, etc. The tendency was rather constant and the percentage of such cases were close to 80% indoor and 20% outdoor. This picture is similar to the situation in other countries [24–26]. Another observation considers the relationship between age of patients and circumstances of hip fracture (indoor or outdoor). The patients that had fallen outside the home were younger (*p* < 0.01). Older individuals are mostly weaker, spent more time at home because of sarcopenia and fall even during daily activities. Evidently, we could conclude that more attention must be paid to the environment (e.g., activity at home, housekeeping, setting household appliance, and light switches, etc) of older patients, since we are face with an ever increasing trend of aging population. First of all, except education, older people should be rehabilitated with special protocols to improve muscular strength [27, 28].

The outdoor fractures are more frequent in men (29.5%) than in women (15.2%) and this difference is statistically significant (*p* < 0.01). Women are probably more careful and because of polish model of lifestyle spent more time at home. The ratio describing the quotient of % fracture indoor and outdoor was the same when we divided patients on place of residence as either urban or rural to be 81–19%, respectively. The average age of the patients with fracture was similar in both groups. The differences were not statistically significant. We concluded that the lifestyle and behaviour in both groups are similar.

In the urban as well as in the rural population, we also observe an increasing trend in fractures. The number of fractures in the observed population was significantly higher in the urban than in the rural area in each study year, evidently by the threefold increase in the urban population. Our study has not revealed the significantly higher rate of hip fracture in the urban or rural areas, similar to the other studies [29–31]. This could be explained by nearness of the areas and in consequence similar lifestyles of their inhabitants, as well as frequent migration. Probably, further observation in larger population is necessary.

According to our estimations, the situation in the future will be, unfortunately, worse. If the trend does not change the increase in next 35 years will be significant. We noted 78 fractures in 2002 (1 patient per 4 or 5 days) and project 390 in 2050 (1 or 2 new patients in every single day). We hope that these estimations will contribute to changing for the betterment of orthopaedic and trauma departments. The problem is projected not only in Poland but could also be extrapolated to other parts of Europe. Due to aging population, similar situation is also expected in the other countries [3, 32–35]. In 2050, the crude rate is projected to be 467.2/100,000 (95% CI 422.0–515.9) for all population and according to Kanis classification, Poland will be in a group of countries with high risk for hip fracture [23].

It is also worth noting that this study has some limitations. Few subjects from the studied region could have also suffered from hip fracture in other part of the country and then the total number of fractures and the rates could be slightly underestimated. Furthermore, some patients could have not been hospitalized due to various reasons. This article was based only on the data from medical documentation. The information on the modifying factors such as diet, physical activity, and fall rate was not available and was not taken into consideration. We have analysed only one district and a projection to whole country on the basis of our study may not express sufficiently reliable number of hip fractures in the whole country.

Finally, the number of fragility hip fractures in Polish men and women aged over 50 years is still relatively low, but the epidemiological situation is getting worse. For over 13 years, we have observed the increasing trend of the total number of hip fractures in both men and women and made a significant projection for the future base on careful detailed analysis and growing trends. Such trend could be explained by fast aging of the Polish population and no specific fragility fracture prevention programs.
